# Δ12-Fatty Acid Desaturase from *Candida parapsilosis* Is a Multifunctional Desaturase Producing a Range of Polyunsaturated and Hydroxylated Fatty Acids

**DOI:** 10.1371/journal.pone.0093322

**Published:** 2014-03-28

**Authors:** Aleš Buček, Petra Matoušková, Hana Sychrová, Iva Pichová, Olga Hrušková-Heidingsfeldová

**Affiliations:** 1 Institute of Organic Chemistry and Biochemistry, Academy of Sciences of the Czech Republic, Prague, Czech Republic; 2 Institute of Physiology, Academy of Sciences of the Czech Republic, Prague, Czech Republic; California Department of Public Health, United States of America

## Abstract

Numerous Δ12-, Δ15- and multifunctional membrane fatty acid desaturases (FADs) have been identified in fungi, revealing great variability in the enzymatic specificities of FADs involved in biosynthesis of polyunsaturated fatty acids (PUFAs). Here, we report gene isolation and characterization of novel Δ12/Δ15- and Δ15-FADs named *Cp*Fad2 and *Cp*Fad3, respectively, from the opportunistic pathogenic yeast *Candida parapsilosis*. Overexpression of *Cp*Fad3 in *Saccharomyces cerevisiae* strains supplemented with linoleic acid (Δ9,Δ12-18:2) and hexadecadienoic acid (Δ9,Δ12-16:2) leads to accumulation of Δ15-PUFAs, *i.e.*, α-linolenic acid (Δ9,Δ12,Δ15-18:3) and hexadecatrienoic acid with an unusual terminal double bond (Δ9,Δ12,Δ15-16:3). *Cp*Fad2 produces a range of Δ12- and Δ15-PUFAs. The major products of *Cp*Fad2 are linoleic and hexadecadienoic acid (Δ9,Δ12-16:2), accompanied by α-linolenic acid and hexadecatrienoic acid (Δ9,Δ12,Δ15-16:3). Using GC/MS analysis of trimethylsilyl derivatives, we identified ricinoleic acid (12-hydroxy-9-octadecenoic acid) as an additional product of *Cp*Fad2. These results demonstrate that *Cp*FAD2 is a multifunctional FAD and indicate that detailed analysis of fatty acid derivatives might uncover a range of enzymatic selectivities in other Δ12-FADs from budding yeasts (Ascomycota: Saccharomycotina).

## Introduction

Unsaturated fatty acids (UFAs) play a key role in maintenance of optimal physical and biological properties of cell membranes [1]. UFAs are biosynthesized from saturated fatty acids by two evolutionary unrelated classes of fatty acid desaturases: soluble fatty acid desaturases, which are expressed exclusively in plant plastids, and membrane-bound fatty acid desaturases (subsequently referred to as FADs), which are widespread in eukaryotes and some prokaryotes [2].

FADs are highly specific towards their fatty acyl substrates (either acyl-CoA or acyl-lipid) and towards the position and geometric configuration of the newly introduced double bond. A range of desaturase regioselectivities dependent on the reference point used by FADs to position the introduced double bond has been described. The main regioselective modes are: (1) the double bond is introduced between specific carbon atoms counted from the carboxy terminus (ΔX) or (2) methyl terminus (ωX) of the fatty acyl substrate, and (3) a subsequent double bond is introduced a specific number of carbon atoms (usually three) counted from the pre-existing double bond (ν+3) [3]. However, these FAD regioselectivities are not mutually exclusive, and Meesapyodsuk *et al.* [4] suggested assigning them primary and secondary modes to more precisely describe FAD regioselectivity. For example, multifunctional FADs preferentially producing Δ9,Δ12-18:2 and Δ9,Δ12-16:2 from Δ9-UFAs but also capable of producing minor amounts of Δ9,Δ12,Δ15-18:3 and Δ9,Δ12,Δ15-16:3 might be termed as v+3 (Δ12), indicating that the primary desaturase regioselectivity is v+3 and the preferred regioselectivity is Δ12. However, this nomenclature may require detailed information on FAD diagnostic substrates and products, which are often not available in the literature. Therefore, we will adhere to nomenclature designating FADs as Δ12- and/or Δ15-FADs.

In fungal species such as *Saccharomyces cerevisiae* or *Candida glabrata*, the main UFAs are Δ9-UFAs. By contrast, in numerous Basidiomycota, Zygomycota and Ascomycota fungal species, such as *Coprinus cinereus* [5], *Mortierella alpina* [6]–[8], *Fusarium moniliforme* [9], *Aspergillus nidulans* [10], *Pichia pastoris* [11], [12], *Kluyveromyces lactis* [13], *Saccharomyces kluyveri* [14], [15] and the majority of *Candida* species [16], Δ12- and Δ15-FADs can desaturate Δ9-UFAs to polyunsaturated fatty acids (PUFAs).

Bifunctional Δ12/Δ15-FADs have been characterized in Ascomycota [4], [9], [10], [17], Basidiomycota [5] and the protozoon *Acanthamoeba castellan* [18]. Additionally, bi- or multifunctional FADs that had previously been identified as FADs with a single desaturase regioselectivity [8], [19] were recently reported [20], [21]. As a result of the similarity between desaturation and hydroxylation reaction pathways, FADs from different kingdoms additionally produce minor amounts of hydroxy fatty acids [22]–[24].

The role of Δ9-FADs in pathogenic *Candida* sp. has been demonstrated in *C. albicans*, in which partial repression of Ole1p Δ9-FAD blocks hyphae and chlamydospore formation [25]. In *C. parapsilosis*, gene deletion of OLE1 impaired invasive growth, pseudohyphae formation and virulence in mice and increased susceptibility to macrophages and various stress factors such as SDS, salts and H_2_O_2_ [26].

The role of PUFAs is more elusive than that of Δ9-UFAs, and PUFAs are probably not essential under all growth conditions. Murayama *et al.* [27] demonstrated that the loss of PUFA production in *C. albicans via* targeted disruptions of the Δ12- and Δ15-FAD genes *Ca*FAD2 and *Ca*FAD3, respectively, did not lead to any changes in growth rate under low temperature, chlamydospore formation, hyphal formation or virulence under the conditions tested. In contrast, the hyphal form of *C. albicans* is enriched in PUFAs compared to the yeast form which suggests a specific role for PUFAs in *C. albicans* morphogenesis [28].

In this study, we isolated the coding regions *Cp*FAD2 and *Cp*FAD3, homologs of fungal Δ12- and Δ15-FADs, respectively, from *C. parapsilosis*, an emerging fungal pathogen [29]. We expressed these FADs in a *Saccharomyces cerevisiae* expression system and identified a broad range of novel polyunsaturated and hydroxylated FA products, including an unusual Δ9,Δ12,Δ15-16:3 with a terminal double bond.

## Methods

### Strains, media and growth conditions

*Saccharomyces cerevisiae* strain BY4741 (MATa his3Δ1 leu2Δ met15Δ ura3Δ; EUROSCARF, Germany) was cultivated in liquid medium lacking uracil (YNBglc-U: 0.67% yeast nitrogen base without amino acids, 2% glucose, supplemented with Brent supplement mix without uracil according to the manufacturer's instructions). In cultivation media, 2% glucose was used as the carbon source. Heterologous protein expression was induced in YNB-U media containing 2% galactose as the sole carbon source (YNBgal-U). When indicated, media were supplemented with 0.5 mM linoleic acid (Sigma-Aldrich), 0.25 mM hexadecadienoic acid (*Z*9,*Z*12-16:2; Larodan), 1% tergitol and 0.65 M NaCl. *C. parapsilosis* (clinical isolate P-69 obtained from the mycological collection of the Faculty of Medicine, Palacky University, Olomouc, Czech Republic) was cultivated prior to lipid extraction in YNBglc medium at 37°C until the culture reached a stationary growth phase.

### Sequence and phylogenetic analysis

The assembly of shotgun reads of *C. parapsilosis* genome publicly available in Candida Genome database was searched using the BLAST tool (http://www.candidagenome.org/cgi-bin/compute/blast_clade.pl) with previously characterized Δ12- and Δ15-FADs as a query.

Amino acid sequences of FADs were aligned using the Muscle algorithm [30], and the phylogeny of FADs was reconstructed with MEGA5 software [31] by neighbor-joining method with calculated bootstrap support from 1,000 bootstrap replicates as a measure of statistical reliability. Desaturase topology was predicted using the programs TMHMM 2.0 [32] and HMMTOP [33]. Pairwise sequence alignment was performed using the EMBOSS Needle web-based tool (http://www.ebi.ac.uk/Tools/psa/emboss_needle/).

Known FAD sequences were retrieved from GenBank. The FAD sequences reported here were deposited into GenBank under accession numbers FN386265 and FN386266.

### Heterologous expression in *Saccharomyces cerevisiae*

Total cellular DNA was extracted from *C. parapsilosis* strain CP-69 and served as a template for PCR amplification of the *Cp*FAD2 coding region using the following primers: *Cp*FAD2-5′ (CTG GAG CTC *ATG* TCT TCA GCC ACA ACT TC) and *Cp*FAD2-3′ (CTG CTC GAG *TTA* TTT CTC TTT CTT TGG GT) (*Sac*I and *Xho*I restriction sites are underlined; start and stop codons are italicized). The *Cp*FAD3 coding region was amplified using the primers *Cp*FAD3-5′ (CAG GAG CTC *ATG* AGT ACG GTC CAT GCA TC) and *Cp*FAD3-3′ (CTG CTC GAG *CTA* GTT TCT TGG TTT GAC AC). The resulting fragments were cloned into the vector pYES2 (Invitrogen) under control of the galactose-inducible *GAL1* promoter, generating plasmids *Cp*FAD2-pYES and *Cp*FAD3-pYES. The constructs were verified by sequencing.

*S. cerevisiae* strain BY4741 was transformed with *Cp*FAD2-pYES, *Cp*FAD3-pYES and empty pYES2 plasmid by electroporation. The resulting *Cp*FAD2, *Cp*FAD3 and Empty yeast strains were selected on YNBglc-U agar plates. Single colonies were inoculated into 20 ml of YNBglc-U and cultivated in a rotary shaker (30°C, 220 RPM). At the early stationary phase, yeast cells were harvested by centrifugation (3 min, 1000 g, 20°C), washed with 20 ml of YNBgal-U and transferred into 20 ml of fresh YNBgal-U (optionally supplemented with linoleic acid, hexadecadienoic acid and tergitol). Yeast strains were cultivated for 72 h.

### Lipid extraction and fatty acid methyl ester preparation

Yeast cells were harvested and total cellular lipids were extracted as described by Buček *et al.* [34], and fatty acid methyl esters (FAMEs) were prepared according to Matoušková *et al.* [35]. The resulting FAMEs were extracted with hexane (600 μl), and the extracts were analyzed by gas chromatography coupled to mass spectrometry (GC/MS) using the conditions described below. The relative abundances of individual fatty acids were calculated from the peak areas in GC/MS total ion current chromatograms.

### DMOX derivatization

Fatty acid 4,4-dimethyloxazoline (DMOX) derivatives were prepared from FAME extracts according to Fay and Richli [36]. Briefly, hexane solvent was evaporated under a stream of nitrogen, and FAMEs were heated at 180°C overnight with 0.5 g 2-amino-2-methylpropanol. After cooling, the DMOX derivatives were dissolved in 5 ml dichloromethane and washed three times with 2 ml distilled water. The dichloromethane phase was dried over anhydrous MgSO_4_, evaporated under a stream of nitrogen and redissolved in hexane. DMOX derivatives were analyzed using GC/MS under the conditions described below and identified by comparing their mass spectra to previously described spectra and using inferred empirical rules [37].

### TMS derivatization

Hydroxy FAs were analyzed in the form of their trimethylsilyl (TMS) derivatives by treating the FAME extracts with excess *N*,*O*-bis(trimethylsilyl)acetamide (Sigma-Aldrich) in acetonitrile (10 min, 40°C), evaporated under a gentle stream of N_2_ and redissolved in chloroform [24]. Freshly prepared TMS derivatives were analyzed using GC/MS under the conditions described below, and their mass spectra and retention behavior were compared to the previously published mass spectra of FAME TMS derivatives [24] and to that of a TMS derivative prepared from 1 mg methyl ricinoleate (methyl 12-hydroxyoleate, Sigma-Aldrich).

### GC/MS analysis

The FAME extracts and respective DMOX and TMS derivatives were analyzed with a 7890A gas chromatograph coupled to a 5975C mass spectrometer, equipped with electron ionisation and quadruple analyser (Agilent Technologies, Santa Clara, CA, USA) using DB-5MS or DB-WAX capillary columns (both J&W Scientific, Folsom, CA, USA; 30 m×0.25 mm, film thickness 0.25 mm); the electron ionisation was set at 70 eV.

Conditions for the analysis of FAMEs and DMOX derivatives were as follows: carrier gas, He: 1 ml/min; 10∶1 split ratio, injection volume 2 μl; injector temperature 220°C; thermal gradient 140°C to 245°C at 3°C/min, then at 8°C/min to 280°C and temperature held for 5 min. The temperature program was terminated at 245°C and held at this temperature for 10 min when the DB-WAX column was used. The TMS derivatives were analyzed on a DB-5MS column using the following thermal gradient: 50°C to 140°C at 10°C/min, then 1°C/min to 198°C, 3°C/min to 320°C and temperature held for 3 min.

## Results

### Sequence and phylogenetic analysis of *Cp*FAD2 and *Cp*FAD3 open reading frames

In the genome database of *C. parapsilosis* [38], we found two open reading frames (ORFs) homologous to fungal FAD2 and FAD3 desaturases and termed them *Cp*FAD2 and *Cp*FAD3, respectively. *Cp*Fad2 shares high amino acid (aa) sequence identity with *C. albicans* Δ12-FAD (80%) [27] and other Δ12-FADs from budding yeasts (Saccharomycotina) (>59%). *Cp*Fad2 shares lower sequence identity with Δ12-FADs from filamentous Ascomycota (Pezizomycotina) (>44%), Basidiomycota (>33%) and Zygomycota (>32%). *Cp*FAD3 shares high sequence identity (79%) with Δ15-FAD from *C. albicans* and with Δ15-FADs from other budding yeasts (61%). *Cp*FAD2 also shares lower sequence identity with bifunctional Δ15>Δ12-FADs from filamentous Ascomycota (>36%) and Zygomycota *Mortierella alpina* (34%).

The *Cp*FAD2 sequence encodes a 426-aa putative polypeptide; *Cp*FAD3 encodes a 432-aa putative polypeptide. *Cp*Fad2 and *Cp*Fad3 share 57% identity and 74% similarity and contain up to six predicted transmembrane helices and a tripartite conserved histidine-rich motif HX_3–4_H, HX_2–3_HH, (H/Q)X_2–3_HH, which indicates that these proteins belong to a non-heme iron enzyme family [3] (Fig. S1).

**Figure 1:**
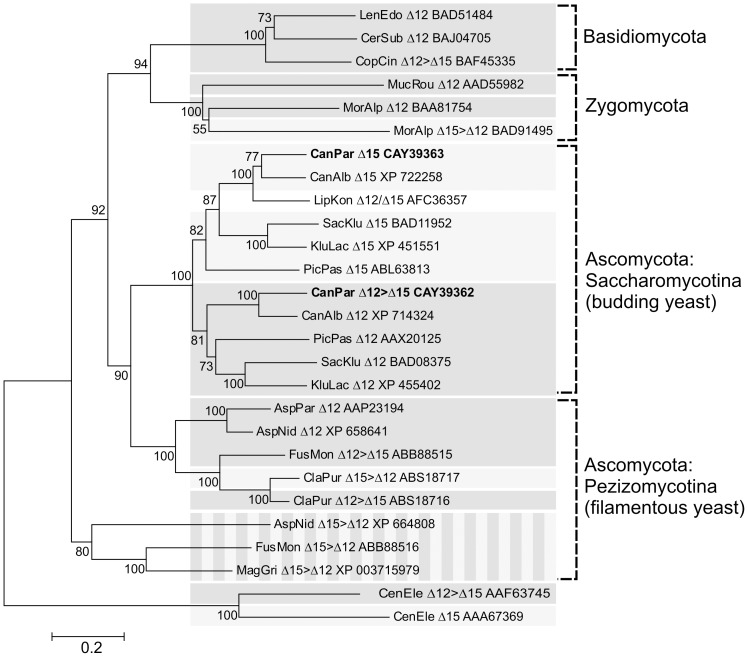
Phylogenetic tree of fungal Δ12- and Δ15-FADs. The species name is abbreviated and followed by experimentally determined desaturase regioselectivity and GenBank protein accession number. For multifunctional FADs, regioselectivity is indicated by “>” (e.g., Δ12>Δ15 indicates a preference for Δ12-desaturation) if available in the literature. Dark grey background denotes Δ12-FADs and Δ12>Δ15-FADs, light grey background denotes Δ15-FADs and Δ15>Δ12-FADs and striped background highlights a clade of bifunctional Δ15>Δ12-FADs. *Cp*FAD2 and *Cp*FAD3 are highlighted in bold. Δ12-FAD and Δ15-FAD from the nematode *Caenorhabditis elegans* were added as an outgroup.

In the reconstructed phylogenetic tree of functionally characterized fungal Δ12- and Δ15-FADs, all FADs cluster into clades according to the class or subphylum of the source organism, with the exception of FADs from the filamentous yeasts *Aspergillus nidulans*, *Fusarium moniliforme* and *Magnaporthe grisea*, which create a group of multifunctional Δ15/Δ12 FADs [9]. Within the clades, FADs cluster generally into “Δ12-FAD” subclades and “Δ15-FAD” subclades. *Cp*Fad2 clusters within a subclade of strictly monofunctional Δ12-FADs, whereas *Cp*Fad3 belongs to a subclade of Δ15-FADs (Fig. 1.).

### Functional characterization of C*p*FAD2 and C*p*FAD3 in *S. cerevisiae*

We designed two pairs of specific primers to amplify the *Cp*FAD2 and *Cp*FAD3 ORFs, and we obtained a single amplicon for each primer pair from genomic DNA isolated from *C. parapsilosis*. Sequencing of *Cp*FAD2 and *Cp*FAD3 confirmed that the nucleotide sequences of the amplified coding regions are identical to those obtained from the *C. parapsilosis* genome database.

The sequence comparison of *Cp*Fad2 and *Cp*Fad3 with previously characterized FADs strongly suggests that *Cp*Fad2 is a Δ12-FAD, whereas *Cp*Fad3 is a Δ15-FAD. However, the homology-based functional annotation of FADs may fail to predict FAD substrate specificity [39] or regioselectivity [14]. To experimentally determine the specificities and regioselectivities of *Cp*Fad2 and *Cp*Fad3, we cloned their coding regions into the pYES2 vector under control of a galactose-inducible promoter. We transformed the resulting plasmids into *S. cerevisiae*, generating the *Cp*FAD2 and *Cp*FAD3 strains.

The total lipidic extracts of galactose-induced *Cp*FAD2 and *Cp*FAD3 strains were transesterified, and the resulting fatty acid methyl esters (FAMEs) were compared to the FAME extract from a control strain transformed with an empty plasmid (Empty strain).

**Figure 2:**
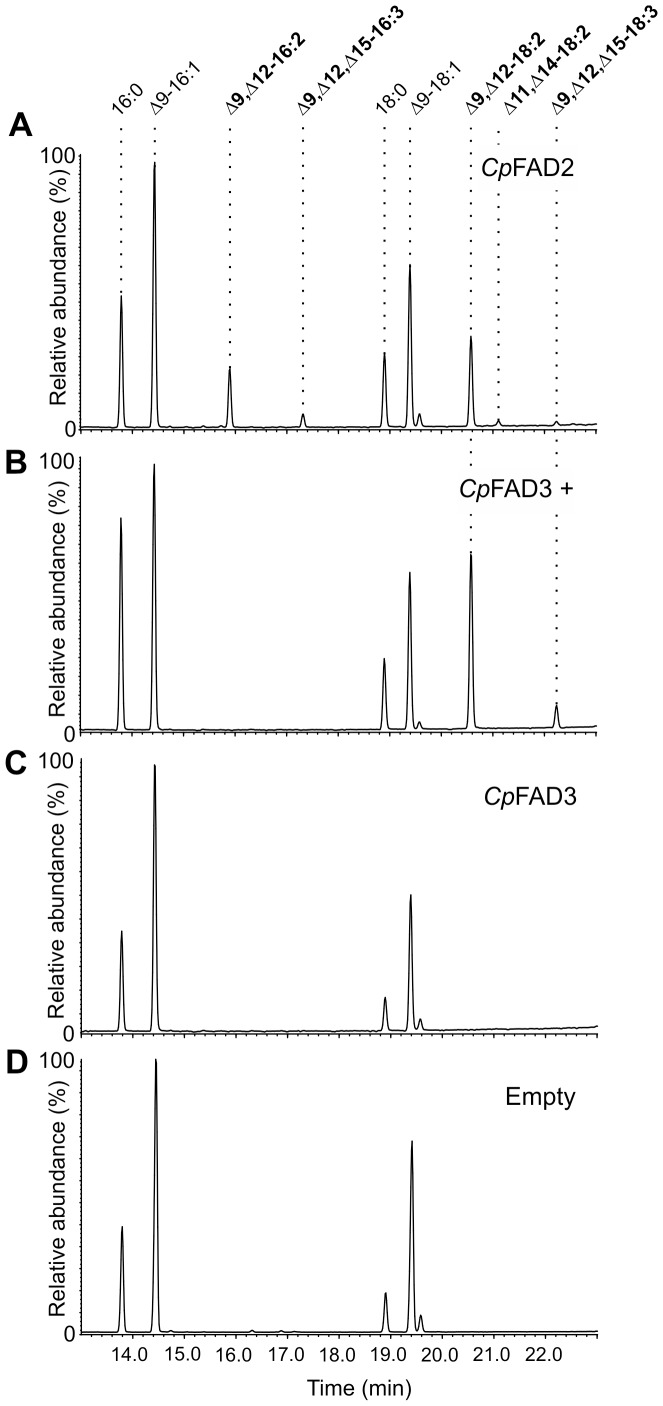
GC chromatograms of FAME extracts from *S. cerevisiae* strains. Analysis of extracts from (**A**) strain expressing *Cp*Fad2, (**B**) strain expressing *Cp*Fad3 grown in media supplemented with 0.5 mM linoleic acid and 1% tergitol, (**C**) strain expressing *Cp*Fad3 and (**D**) strain bearing empty pYES2 plasmid.

**Figure 3:**
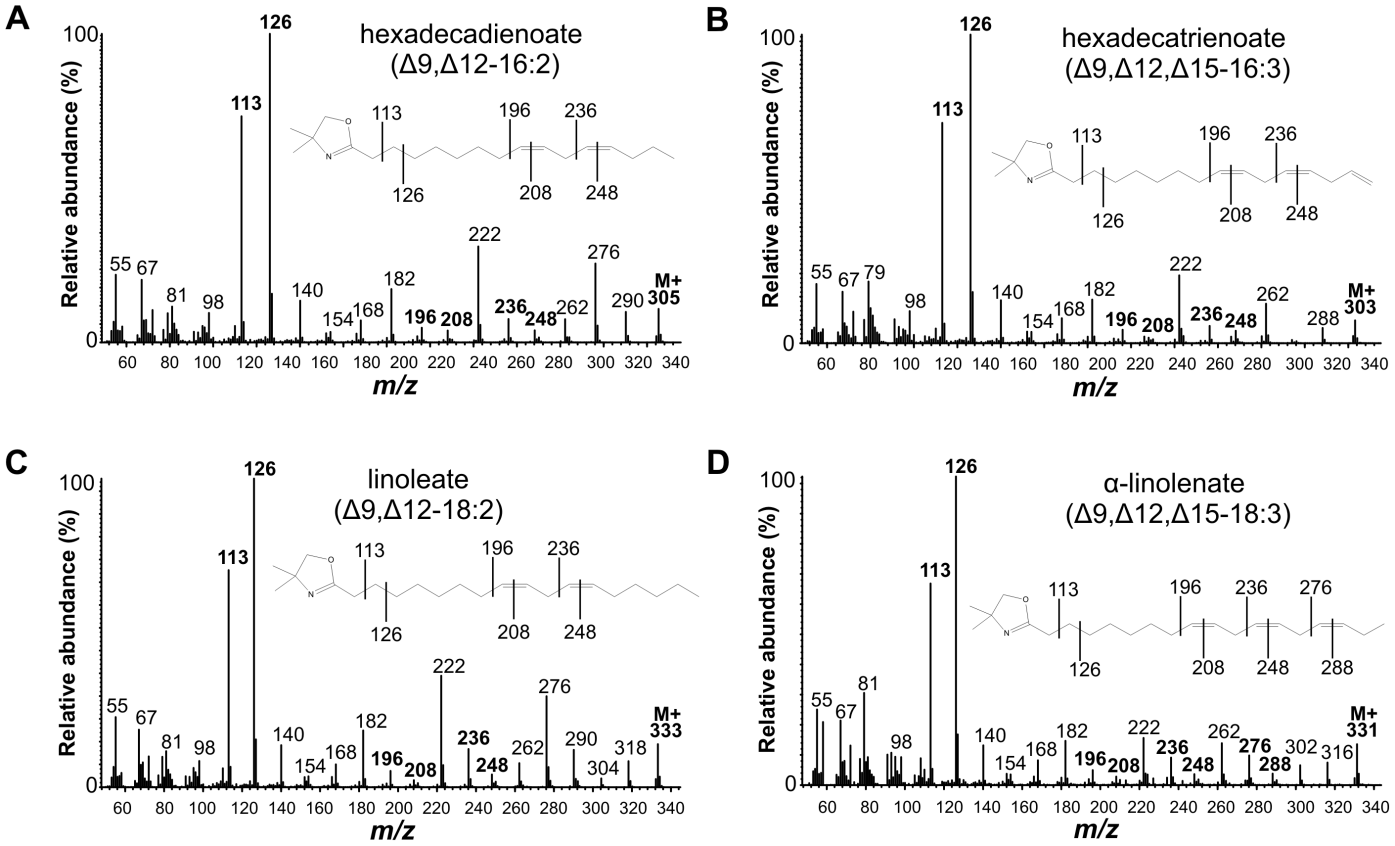
Mass spectra of DMOX derivatives of PUFAs detected in FAME extracts from *Cp*FAD2 and *Cp*FAD3 yeast strains. (**A**) Δ9,Δ12-16:2; (**B**) Δ9,Δ12,Δ15-16:3; (**C**) Δ9,Δ12-18:2; (**D**) Δ9,Δ12,Δ15-18:3. Characteristic fragments are highlighted and fragmentation patterns of DMOX derivatives are shown.

In the FAME extract from the induced *Cp*FAD2 strain, we detected multiple PUFAs that were not present in the FAME extract from the control strain (Fig. 2 and Table 1). The double bond position of all detected PUFAs was confirmed by the presence of characteristic MS fragment ions of DMOX derivatives (Fig. 3). In the *Cp*FAD2 strain, the most abundant novel PUFAs were linoleic acid (Δ9,Δ12-18:2; 11.88% ± 0.99%) and hexadecadienoic acid (Δ9,Δ12-16:2; 5.25% ± 0.65%). Traces of an octadecadienoic acid isomer with a characteristic 12 atomic mass unit gap between m/z 224 to 236 and 264 to 276 were detected, which is indicative of a Δ11,Δ14 double bond position (Fig. S2). We presume that Δ11,Δ14-18:2 is an elongation product of Δ9,Δ12-16:2. Unexpectedly, we also detected α-linolenic acid (Δ9,Δ12,Δ15-18:3; 0.12% ± 0.11%) in the *Cp*FAD2 strain, which indicates that the α-linolenic acid is synthesized from linoleic acid *via* minor Δ15-desaturase activity of *Cp*FAD2 desaturase (Fig. 2).

**Table 1:** Relative abundances of fatty acids in FAME extracts of total cellular lipids from yeast strains.

	Fatty acid composition (%)				
Fatty acid	Empty	Empty+	*Cp*FAD2	*Cp*FAD3	*Cp*FAD3+
**16:0**	27.22 ± 0.98	24.64 ± 1.01	27.04 ± 0.12	29.32 ± 0.58	25.84 ± 0.56
**Δ9-16:1**	41.99 ± 1.15	5.07 ± 0.06	29.71 ± 0.10	36.56 ± 0.48	3.45 ± 0.74
**Δ9, Δ12-16:2**	n.d.	n.d.	5.25 ± 0.65	n.d.	n.d.
**Δ9, Δ12, Δ15-16:3**	n.d.	n.d.	0.77 ± 0.09	n.d.	n.d.
**18:0**	6.41 ± 0.51	6.21 ± 1.06	8.97 ± 0.30	8.72 ± 0.36	8.23 ± 0.61
**Δ9-18:1**	24.38 ± 1.07	2.72 ± 0.26	16.26 ± 1.41	25.41 ± 0.43	2.74 ± 0.41
**Δ9, Δ12-18:2**	n.d.	61.36 ± 1.99	11.88 ± 0.99	n.d.	52.79 ± 1.15
**Δ11, Δ14-18:2**	n.d.	n.d.	0.35 ± 0.01	n.d.	n.d.
**Δ9, Δ12, Δ15-18:3**	n.d.	n.d.	0.12 ± 0.11	n.d.	6.95 ± 0.99

The relative amount of fatty acids is expressed as a percentage of total fatty acid methyl esters. Strains supplemented with 0.5 mM linoleic acid and 1% tergitol are marked with “+”. Values represent means of three cultivations ± standard deviation. n.d.: FAME not detected.

**Figure 4:**
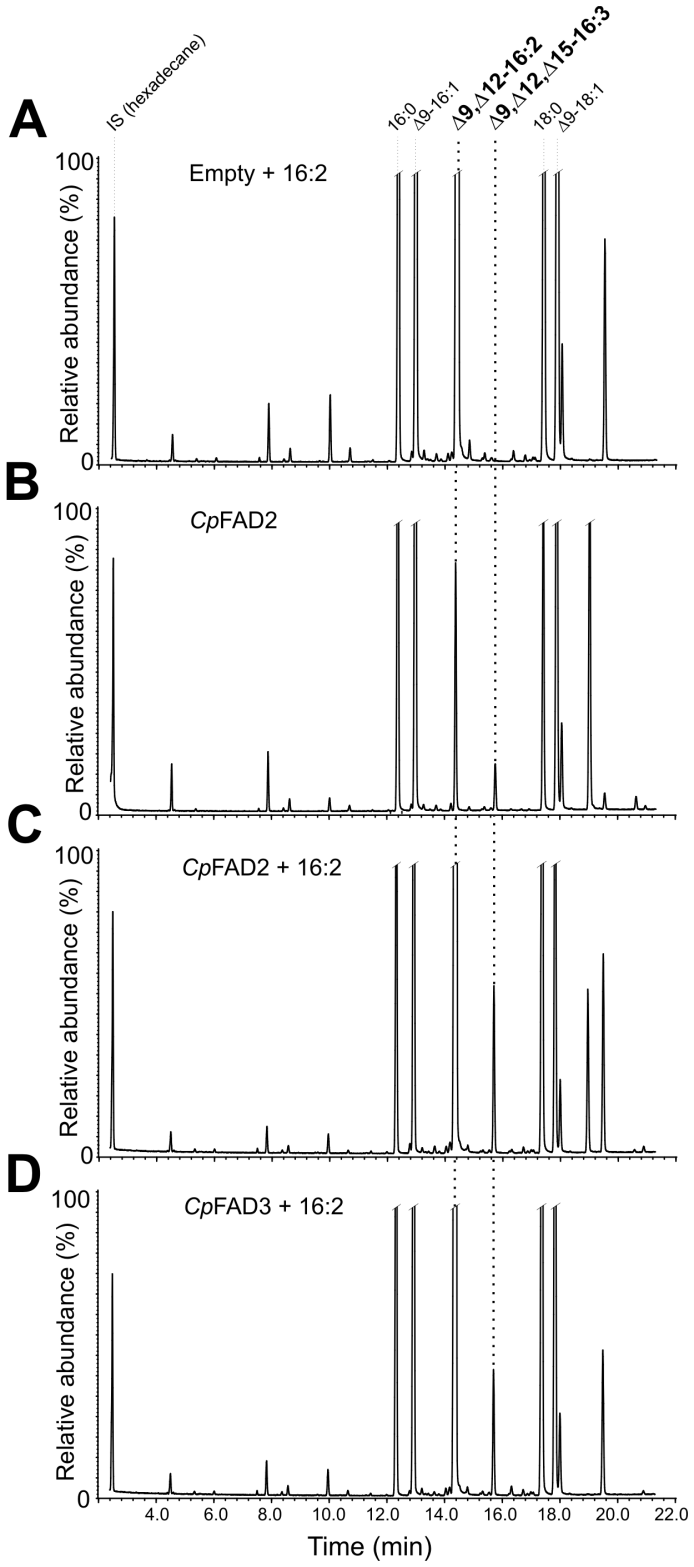
GC chromatograms of FAME extracts from yeasts supplemented with hexadecadienoic acid (Δ9,Δ12-16:2). GC/MS analysis of FAME extracts from (**A**) Empty strain supplemented with hexadecadienoic acid (Δ9,Δ12-16:2), (**B**) *Cp*FAD2 strain, (**C**) *Cp*FAD2 strain supplemented with Δ9,Δ12-16:2 and (**D**) *Cp*FAD3 strain supplemented with Δ9,Δ12-16:2. Hexane containing internal standard (hexadecane at concentration of 20 μg/ml) was used in preparation of all extracts.

Additionally, a hexadecatrienoate (0.77% ± 0.09%) with double bonds in position Δ9 and Δ12 and a third double bond in either the Δ14- or Δ15-position was identified in the *Cp*FAD2 strain (Fig. 2). The DMOX derivatives of FAMEs with terminal double bonds do not produce the characteristic fragmentation pattern exhibiting a 12 atomic mass unit gap [40]. However, we could unambiguously identify the triunsaturated product of *Cp*Fad2 as Δ9,Δ12,Δ15-16:3 with terminal (n-1) double bond by comparing its spectrum to that of Δ9,Δ12,Δ15-16:3 methylester [41] (Fig. S3) and to the mass spectrum of a previously described DMOX derivative of Δ9,Δ12,Δ15-16:3 [18], [21] (Fig. 3B). To confirm the production of Δ9,Δ12,Δ15-16:3 *via* desaturation of Δ9,Δ12-16:2, the *Cp*FAD2 strain was supplemented with Δ9,Δ12-16:2. The increase in relative abundance of Δ9,Δ12,Δ15-16:3 in the supplemented *Cp*FAD2 strain indicated that Δ9,Δ12,Δ15-16:3 is produced by Δ15-desaturation (Fig. 4B, C).

In the *Cp*FAD3 strain, supplementing the cultivation medium with linoleic acid led to production of α-linolenic acid (3.62% ± 0.15%). No polyunsaturated products were detected in the *Cp*FAD3 strain cultivated without PUFA supplementation (Fig. 2 and Table 1). Surprisingly, supplementing the *Cp*FAD3 cultivation medium with Δ9,Δ12-16:2 led to production of Δ9,Δ12,Δ15-16:3 (Fig. 4D). To rule out the possible interference of yeast Δ9-FAD in the metabolism of Δ9,Δ12-16:2, the Empty strain was also supplemented with Δ9,Δ12-16:2. GC/MS analysis confirmed that Δ9,Δ12-16:2 is not desaturated to hexadecatrienoic acid in the Empty strain (Fig. 4A).

To determine the distribution of novel PUFAs in individual lipid classes, the total lipidic extract was analyzed using liquid chromatography with mass spectrometric analysis. The preliminary data indicates that PUFAs are distributed in phospholipids, *i.e.*, phosphatidylserine, phosphatidylethanolamine, phosphatidylcholine, and triacylglycerols (data not shown). We have also investigated the effect of accumulation of PUFAs in the *Cp*FAD2 and *Cp*FAD3 strains on their growth rate and tolerance to alkali-metal cations. When compared to the Empty strain, we observed a decreased growth rate for yeasts expressing *Cp*FAD2 on YNBgal-U agar plates (Fig. S4B). The decreased growth rate of the *Cp*FAD2 strain became even more prominent on YNBgal-U containing 0.65 M NaCl (Fig. S4C). The growth rate of the *Cp*FAD3 strain on YNBgal-U or YNBgal-U supplemented with linoleic acid was unaltered compared to that of the Empty strain (Fig. S4B, D).

### Hydroxylation activity of C*p*Fad2 and C*p*Fad3

**Figure 5:**
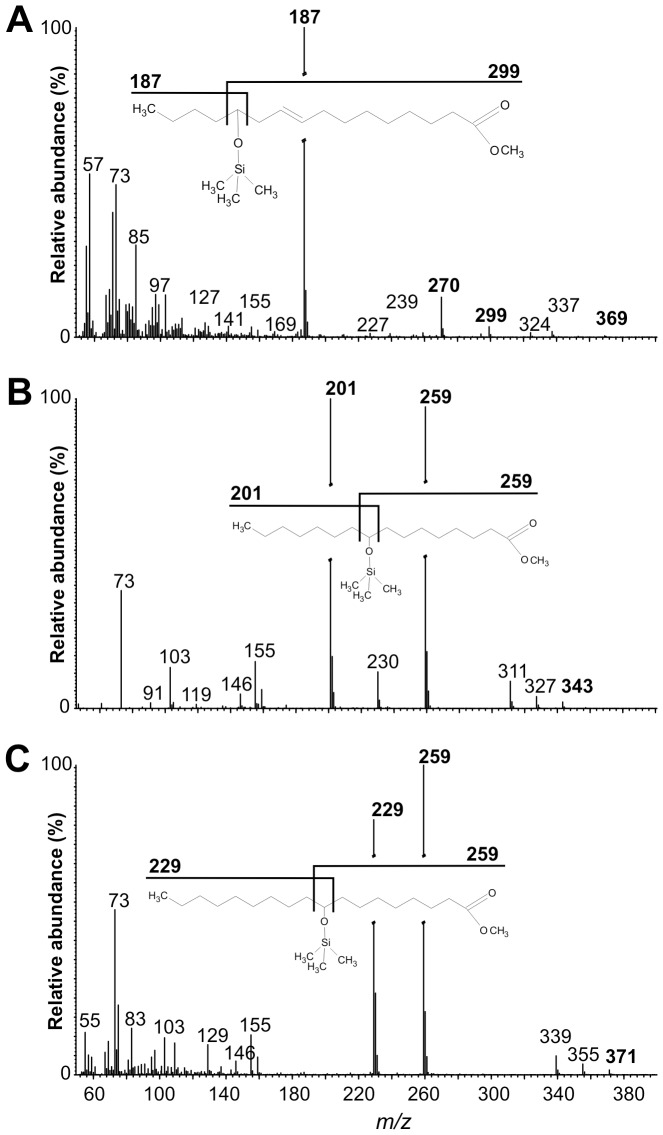
Mass spectra of TMS derivatives of hydroxy fatty acid methyl esters. (**A**) TMS-methyl ricinoleate detected in FAME extracts from *Cp*FAD2 yeast strain. (**B**) TMS- methyl hydroxypalmitate detected in all yeast strains. (**C**) TMS-methyl hydroxystearate detected in all yeast strains. Characteristic fragments are highlighted in bold, and fragmentation patterns of TMS derivatives are shown above the spectra.

To determine whether *Cp*Fad2 and *Cp*Fad3 are capable of FA hydroxylation, we treated the FAME extracts with *N*,*O*-bis(trimethylsilyl)acetamide to convert hydroxy FAMEs to their corresponding trimethylsilyl-FAMEs, which exhibit better chromatographic properties and provide MS fragment ions characteristic of particular TMS group locations [42]. Trace amounts of TMS derivatives of methyl 9-hydroxypalmitic acid (MS fragment ions at m/z 201 and 259) and methyl 9-hydroxystearic acid (MS fragment ions at m/z 229 and 259) were detected in all transformed yeast strains (Fig. 5, S5 and Table 2), providing evidence of the intrinsic Δ9-fatty acid hydroxylase activity of yeast Δ9-FAD Ole1p [22], [24]. Notably, in the *Cp*FAD2 strain, an additional TMS derivative with low abundance (0.10% ± 0.02%) was detected (Table 2). This was determined to be a TMS derivative of methyl ricinoleate (methyl 12-hydroxyoleate) based on its retention time, which was identical to that of TMS derivative of methyl ricinoleate standard, and on the presence of characteristic fragment ions, namely (M-15) ion at m/z 369 and ions at m/z 187, 270 and 299 (Fig. 5A). This observation indicates that *Cp*FAD2 exhibits 12-hydroxylation activity.

**Table 2:** Relative abundances of TMS derivatives of hydroxy FAs in FAME extracts from *Cp*FAD2, *Cp*FAD3 and Empty strains.

	TMS-derivative composition (%)				
TMS-derivative	Empty	Empty+	*Cp*FAD2	*Cp*FAD3	*Cp*FAD3+
**TMS-methy hydroxypalmitate**	0.09 ± 0.01	0.05 ± 0.01	0.10 ± 0.03	0.09 ± 0.01	0.04 ± 0.01
**TMS-methyl ricinoleate**	n.d.	n.d.	0.10 ± 0.02	n.d.	n.d.
**TMS-methyl hydroxystearate**	0.06 ± 0.00	0.02 ± 0.00	0.09 ± 0.00	0.07 ± 0.01	0.02 ± 0.00

The relative amount of FAME-TMS derivatives is expressed as a percentage of total fatty acid methyl esters. Strains supplemented with 0.5 mM linoleic acid and 1% tergitol are marked with “+”. Values represent means of three cultivations ± standard deviation. n.d.: TMS-derivative not detected.

### PUFA content in *C. parapsilosis*

Although the relative abundance of linoleic acid in *C. parapsilosis* (22.18%) was higher than that in the *Cp*FAD2 *S. cerevisiae* strain (11.88%), we detected only trace amounts of Δ9,Δ12-16:2 (0.04%) in *C. parapsilosis* in contrast to moderately abundant Δ9,Δ12-16:2 (5.25%) in the *Cp*FAD2 strain. Notably, Δ9,Δ12,Δ15-16:3 was completely absent in *C. parapsilosis* under the conditions tested (Table 3). No additional PUFAs were detected under low cultivation temperatures (25°C or 30°C) (data not shown).

**Table 3:** Relative abundance of fatty acids in FAME extracts from *C. parapsilosis*.

	Fatty acid composition (%)
Fatty acid	*C.parapsilosis*
**16:0**	12.54 ± 0.07
**Δ9-16:1**	3.38 ± 0.04
**Δ9,Δ12-16:2**	0.05 ± 0.01
**Δ9,Δ12,Δ15-16:3**	n.d.
**18:0**	4.31 ± 0.09
**Δ9-18:1**	54.96 ± 0.40
**Δ9,Δ12-18:2**	22.89 ± 0.15
**Δ9,Δ12,Δ15-18:3**	1.90 ± 0.24

The relative amount of fatty acids is expressed as a percentage of total fatty acid methyl esters. Values represent means of three cultivations ± standard deviation. n.d.: FAME not detected.

## Discussion

Fungal FAD research is largely motivated by the search for novel FADs that could be used in metabolic engineering of microorganisms to produce PUFAs and other valuable UFAs on an industrial scale [43], [44]. Additionally, desaturation, as a part of fungal fatty acid metabolism, has been shown to play a crucial role in the growth and morphogenesis of pathogenic yeast species in plants and humans. Therefore, FADs have been suggested as potential targets for antifungal drugs [25]–[27], .

Nguyen *et al*. [26] described the role of Δ9-FAD in the pathobiology of *C. parapsilosis*, a fungal pathogen that forms persistent biofilms on implanted medical devices such as catheters [29]. In addition to saturated and monounsaturated fatty acids, *C. parapsilosis* cellular lipids contain a major fraction of PUFAs, suggesting activity of Δ12- and Δ15-FADs [16]. In this study, we identified and characterized *Cp*Fad2 and *Cp*Fad3 desaturases responsible for production of PUFAs in *C. parapsilosis*.

Sequence analysis of *Cp*Fad2 and *Cp*Fad3 using TMHMM 2.0 and HMMTOP topology prediction algorithms indicated the presence of more than four transmembrane helices. The proposed topology, however, does not satisfy the requirement of arrangement of conserved histidine motifs on a common, cytosolic ER membrane face [47], [48] (Fig. S1). Therefore, the third and fourth transmembrane helix likely represents a hydrophobic region peripherally associated with an ER membrane. This topology was previously proposed by Hoffmann *et al.* for Δ12-FAD and a bifunctional Δ12/Δ15-FAD from *Aspergillus nidulans* [10]. Alternatively, some of the additional TM helices might be correctly predicted and play a role in binding the acyl-lipid substrate, as hypothesized by Diaz *et al.* [49].

Several identified FADs can produce unusual terminally unsaturated Δ9,Δ12,Δ15-16:3 fatty acids, including FADs from the protozoon *Acanthamoeba castellanii* [18], the basidiomycete fungus *Coprinus cinereus* [5], the ascomycete filamentous fungus *Claviceps purpurea* [4], and the ascomycete budding yeast *Lipomyces kononenkoae* [17]. Δ9,Δ12,Δ15-16:3 also has been identified as an initially undiscerned product of Δ15-FAD from the zygomycete fungus *Mortierella alpina* [21] and Δ12-FAD from the nematode *Caenorhabditis elegans* [20].

Previously, Δ9,Δ12,Δ15-16:3 was not detected as a product of *Ca*Fad2 heterologously expressed in *S. cerevisiae* under control of the *GAL1* promoter. *Ca*Fad2 is a Δ12-FAD from *C. albicans*, which can produce Δ9,Δ12-16:2 and Δ9,Δ12-18:2 fatty acids [27]. However, the presence of Δ9,Δ12,Δ15-16:3 might be easily missed due to its overall low abundance or due to the low abundance of the molecular ion at *m/z* 264 and low abundance of fragments at *m/z* 74 and 87 characteristic of FAMEs (Fig. S3).

Production of α-linolenic, hexadecatrienoic (Δ9,Δ12,Δ15-16:3) and ricinoleic acid (12-hydroxyoleate) by *Cp*Fad2 suggests that *Cp*Fad2 is a bifunctional Δ12/Δ15-FAD. The Δ15-desaturase activity of Δ12-FAD from budding yeast (Saccharomycotina) has been reported only by Yan *et al.* [17], and the hydroxylase activity has not been investigated. Damude *et al.* suggested that Δ15-FADs independently evolved from ancestral Δ12-FADs multiple times [9]. The facile evolutionary shift between Δ12- and Δ15-FAD regioselectivities was further supported by the effect of site-directed mutagenesis of a single amino acid residue [4] and by domain-swapping experiments [4], [10]. Analogously, one to four amino acid mutations were found to be sufficient to convert plant Δ12-FAD into a bifunctional Δ12-FAD/hydroxylase [22], [50]. Based on these observations and our current results, we hypothesize that minor Δ15-desaturase regioselectivity and hydroxylase activity might be present in numerous functionally characterized Δ12-FADs from budding yeasts, including *Ca*Fad2 from *C. albicans* [27]. The fact that minor PUFA and hydroxylated products were rarely detected in previously described Δ12-FADs from budding yeast might be caused by a low level of desaturase expression under the experimental conditions, by a low level of accumulation of PUFAs and hydroxylated fatty acids or by the low sensitivity of the FAME analysis procedure. The reason underlying the absence of specific Δ15-hydroxylated FAs in *Cp*FAD3 strain supplemented with linoleic acid might be the absence of *Cp*Fad3 Δ15-hydroxylase activity and/or the overall low enzymatic activity of *Cp*Fad3 compared to *Cp*Fad2 in our expression system.

Based on the range of observed desaturase specificities of *Cp*Fad2 [its capability of introducing a double bond at the Δ15 (ω1) position in Δ9,Δ12,Δ15-16:3 and Δ15(ω3) position in Δ9,Δ12,Δ15-18:3 and the lack of Δ9,Δ15-18:2 products], we propose that the primary regioselective mode of *Cp*Fad2 is ν+3 and the secondary mode is Δ12. This would imply, in accordance with the FAD regioselectivity classification described by Meesapyodsuk *et al.* [4], that the preexisting double bond serves as a reference point for positioning of the introduced double bond and that the Δ12-position is preferred over the Δ15-position.

The *Cp*FAD3 strain produced Δ9,Δ12,Δ15-18:3 and Δ9,Δ12,Δ15-16:3 when supplemented with Δ9,Δ12-18:2 and Δ9,Δ12-16:2 PUFAs, respectively. The complete absence of novel PUFAs in the *Cp*FAD3 strain cultivated without addition of linoleic acid or hexadecadienoic acid (ω6 substrates) indicates that *Cp*FAD3 cannot desaturate the naturally present Δ9-FAs, in contrast to, for example, ω3-FADs from *Caenorhabditis elegans* [51] or *Saccharomyces kluyveri* [52]. Together, this data indicates that *Cp*Fad3 exhibits Δ15-regioselectivity requiring a preexisting Δ12-double bond and is capable of introducing a terminal double bond.

Overexpression of Δ12- and Δ15(ω3)-FADs in *S. cerevisiae* provides a tool to study the influence of PUFAs on yeast physiology [19], [53]–[55]. The PUFAs were present in phospholipid fraction of the *Cp*FAD2 and *Cp*FAD3 strain supplemented with linoleic acid, suggesting that they might influence the membrane properties and the yeast phenotype. However, we did not observe any increase in tolerance to NaCl, which has been observed in yeast strains producing PUFAs [55]. A decreased growth rate of yeasts heterologously expressing Δ12-FADs was previously attributed to impairment of tryptophan uptake [54]. Although we employed the BY4741 yeast strain which is not auxotrophic for tryptophan, the modified structure and physical properties of yeast cell membranes containing PUFAs might affect the properties of numerous cell membrane proteins and result in a decreased growth rate.

In *C. parapsilosis*, the low amount or complete absence of C16 PUFAs and low amount of palmitoleic acid is in general agreement with the results of a previous study by Moss *et al.* [16] that describes the fatty acid composition of various *Candida* species. Together, these data suggests that in *Candida* species, C16 PUFAs do not accumulate. The low content of C16 PUFAs might be a consequence of a low amount of their precursor, palmitoleic acid. Alternatively, overexpression of *Cp*Fad2 under control of the *GAL1* promoter might result in higher expression and therefore higher desaturase activity of *Cp*Fad2 in *S. cerevisiae*, as compared to the activity of *Cp*Fad2 in *C. parapsilosis*.

Taken together, this study provides further evidence that, despite the growing database of functionally characterized FADs, detailed characterization of FADs by mass spectrometry analysis of fatty acid derivatives can reveal surprising desaturase specificities that cannot be inferred solely from sequence comparisons.

## Supporting Information

Figure S1 **Amino acid sequence alignment of** ***Cp*** **Fad2 and** ***Cp*** **Fad3.** Three conserved histidine-rich regions (H1–H3) are marked by boxes. Predicted transmembrane domains for *Cp*Fad2 and *Cp*Fad3 are indicated by bars above or below the sequence, respectively. The consensus transmembrane region predicted by both HMMTOP and TMHMM 2.0 algorithms are indicated by solid bars; transmembrane regions predicted by only one algorithm are indicated by dashed bars. Identical residues are indicated by a black background, similar residues by a grey background.
(TIF)

Figure S2 **Mass spectra of DMOX derivative of Δ11,Δ14-18:2-methylester detected in FAME extract from** ***Cp*** **FAD2 yeast strain.** Characteristic fragments are highlighted, and the fragmentation pattern of the DMOX derivative is shown above the spectra.
(TIF)

Figure S3 **Mass spectra of Δ9,Δ12,Δ15-16:3-methylester identified in FAME extract from** ***Cp*** **FAD2 yeast strain.**
(TIF)

Figure S4 **Comparison of growth rates of** ***Cp*** **FAD2,** ***Cp*** **FAD3 and Empty yeast strains.** Yeast suspensions were spotted on (**A**) YNBglc-U agar plates, which repress heterologous protein expression, (**B**) YNBgal-U agar plates, (**C**) YNBgal-U agar plates containing 0.65 M NaCl and (**D**) YNBgal-U agar plates containing 1% tergitol and 0.5 mM linoleic acid. Prior to plating on solid media, yeast strains were grown on YNBglc-U agar plates and incubated overnight at 4°C. The cells then were resuspended in sterile water to an OD_600_ of 1.0. Serial 10-fold dilutions were spotted on the YNB agar plates using a replica plater. The agar plates were incubated at 30°C for 3 days and photographed. Representative images are shown.
(TIF)

Figure S5 **Extracted ion chromatograms of TMS derivatives of hydroxy FAMEs.** TMS derivatives of FAME extracts from the *Cp*FAD2 strain, *Cp*FAD3 strain supplemented with linoleic acid and Empty strain are displayed in ion chromatograms extracted at *m/z* values characteristic for individual TMS-hydroxy FAMEs. (**A**) Ion chromatograms extracted at *m/z* 201 and 259 characteristic for TMS-methyl hydroxypalmitate, (**B**) ion chromatograms extracted at *m/z* 229 and 259 characteristic for TMS-methyl hydroxystearate and (**C**) ion chromatograms extracted at *m/z* 187 and 299 characteristic for TMS-methyl ricinoleate.
(TIF)
