# Supplementary figures and images for: Δ12-Fatty Acid Desaturase from Candida parapsilosis Is a Multifunctional Desaturase Producing a Range of Polyunsaturated and Hydroxylated Fatty Acids
Source: PLoS One. 2014 Mar 28;9(3):e93322. doi: 10.1371/journal.pone.0093322 (PMC3969366; doi:10.1371/journal.pone.0093322)

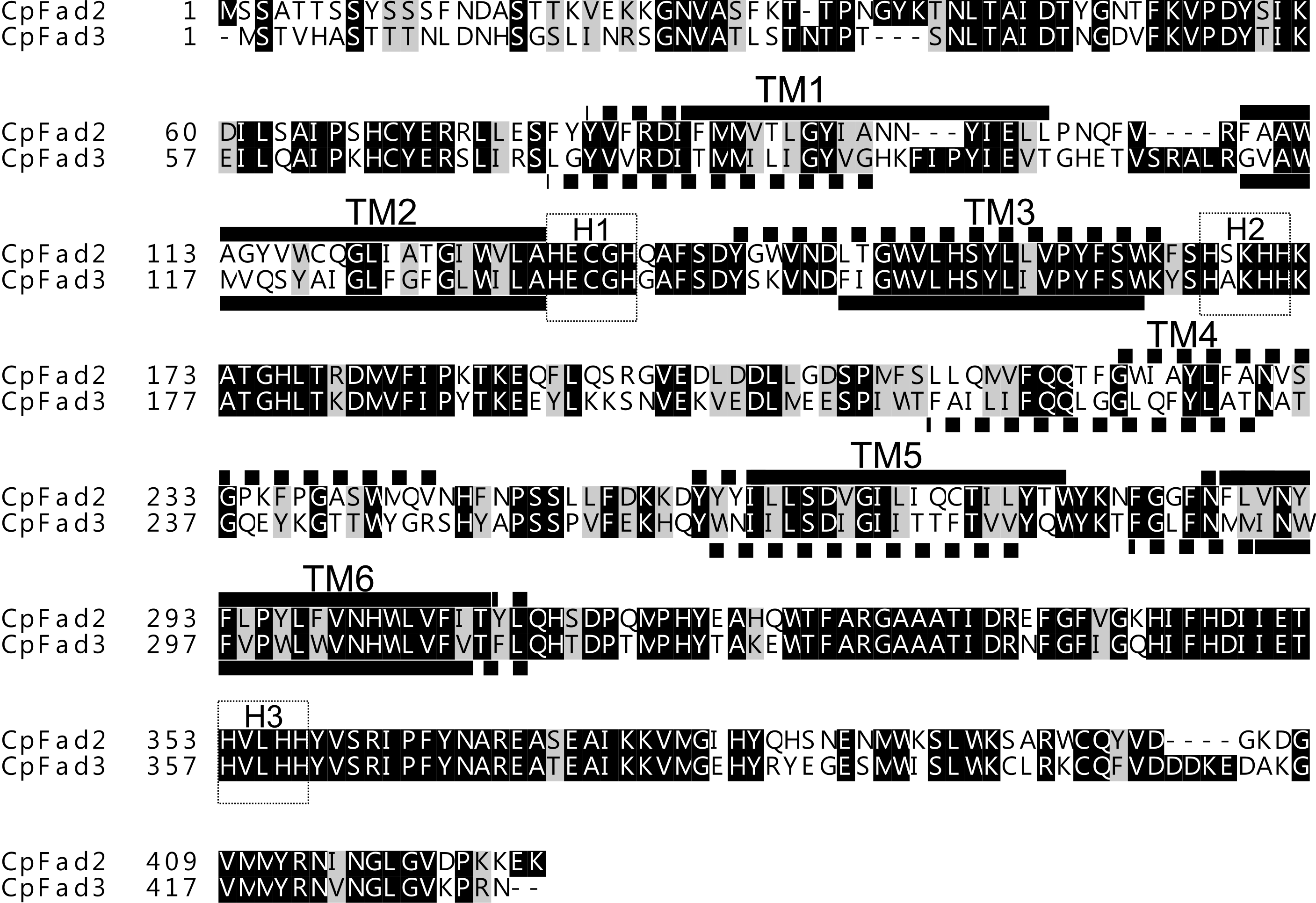

Supplement: Figure S1 — Amino acid sequence alignment of Cp Fad2 and Cp Fad3. Three conserved histidine-rich regions (H1–H3) are marked by boxes. Predicted transmembrane domains for CpFad2 and CpFad3 are indicated by bars above or below the sequence, respectively. The consensus transmembrane region predicted by both HMMTOP and TMHMM 2.0 algorithms are indicated by solid bars; transmembrane regions predicted by only one algorithm are indicated by dashed bars. Identical residues are indicated by a black background, similar residues by a grey background. (TIF) [file pone.0093322.s001.tif]

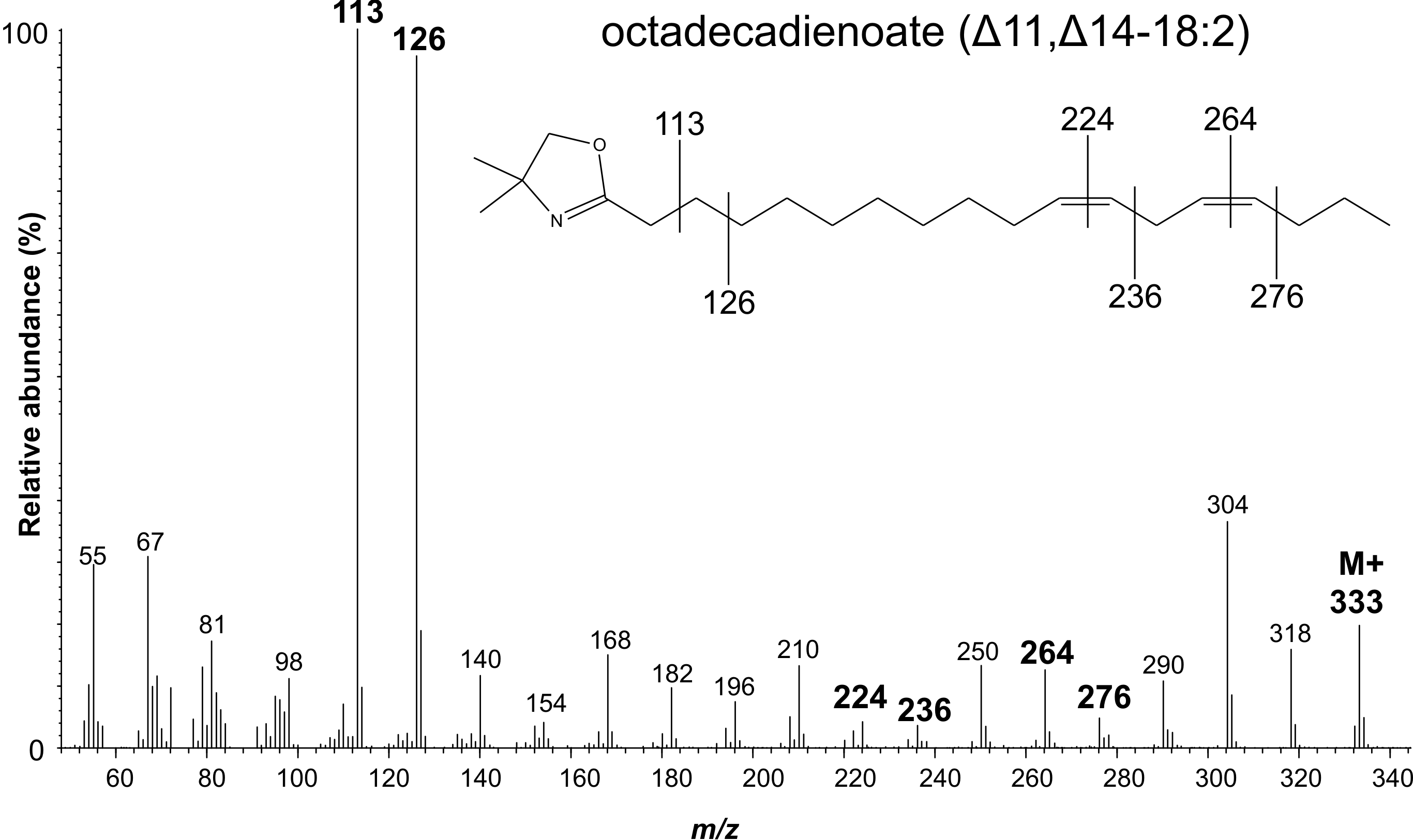

Supplement: Figure S2 — Mass spectra of DMOX derivative of Δ11,Δ14-18:2-methylester detected in FAME extract from Cp FAD2 yeast strain. Characteristic fragments are highlighted, and the fragmentation pattern of the DMOX derivative is shown above the spectra. (TIF) [file pone.0093322.s002.tif]

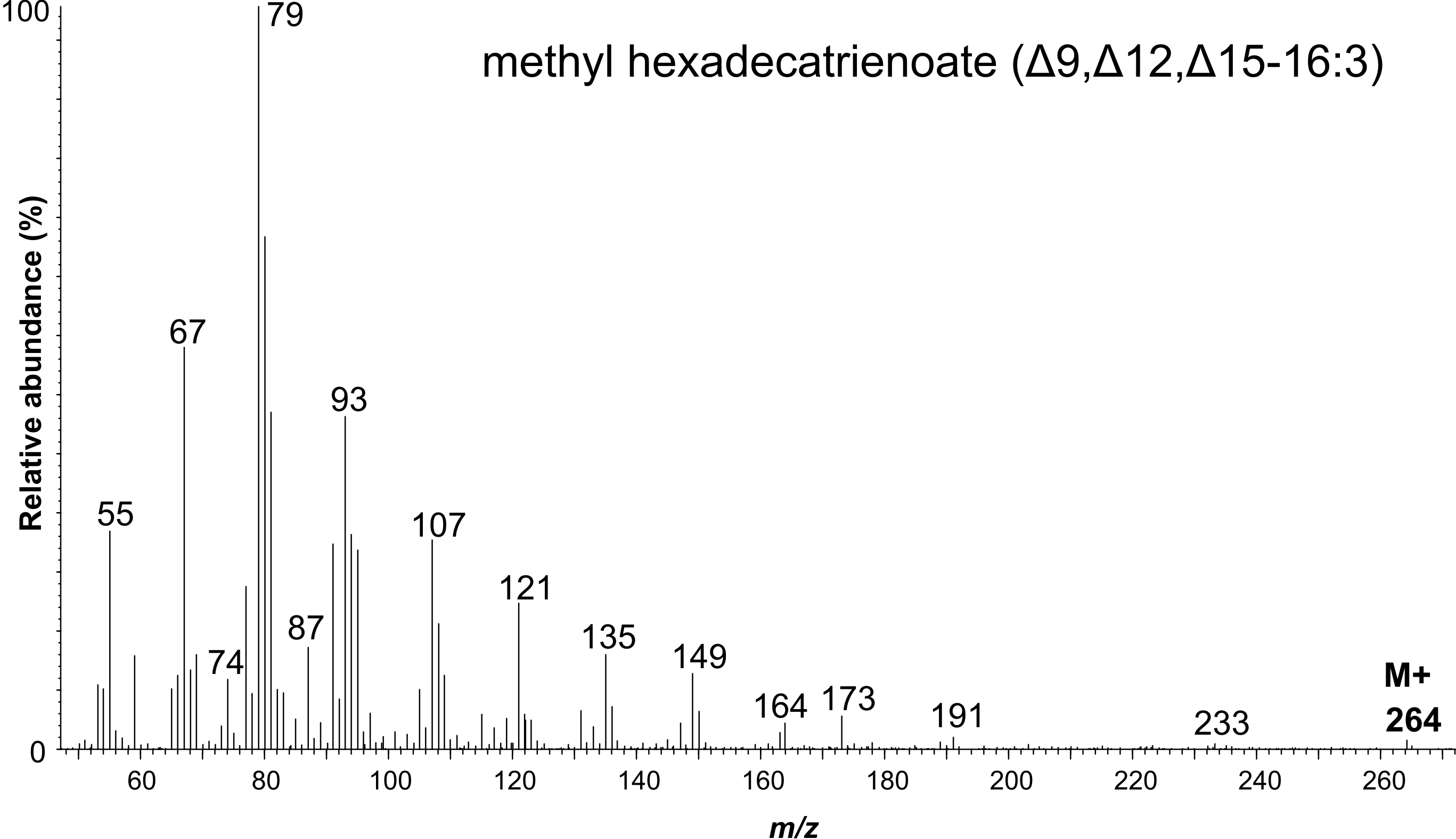

Supplement: Figure S3 — Mass spectra of Δ9,Δ12,Δ15-16:3-methylester identified in FAME extract from Cp FAD2 yeast strain. (TIF) [file pone.0093322.s003.tif]

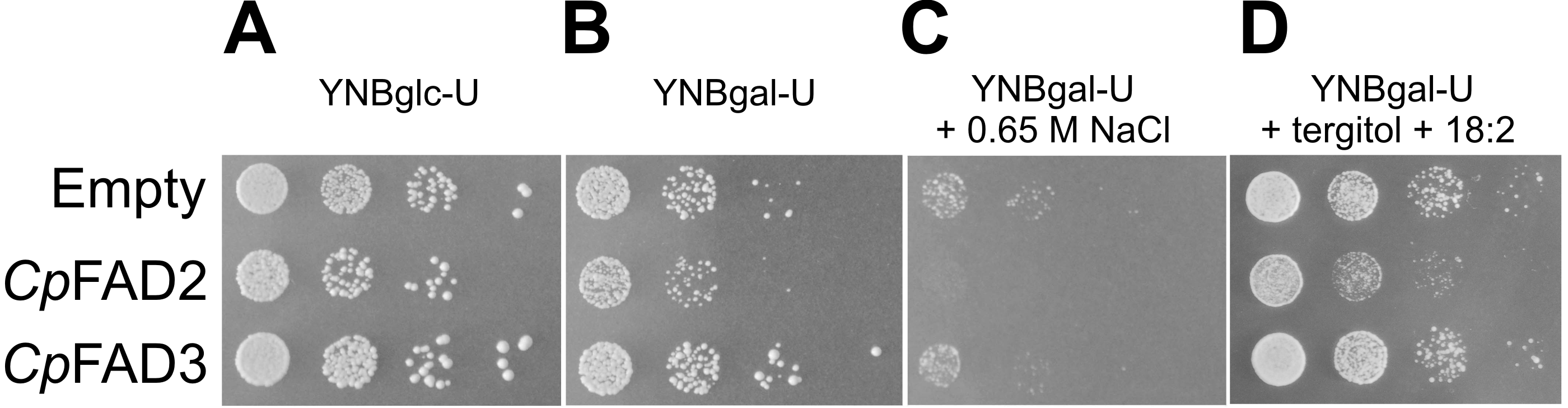

Supplement: Figure S4 — Comparison of growth rates of Cp FAD2, Cp FAD3 and Empty yeast strains. Yeast suspensions were spotted on (A) YNBglc-U agar plates, which repress heterologous protein expression, (B) YNBgal-U agar plates, (C) YNBgal-U agar plates containing 0.65 M NaCl and (D) YNBgal-U agar plates containing 1% tergitol and 0.5 mM linoleic acid. Prior to plating on solid media, yeast strains were grown on YNBglc-U agar plates and incubated overnight at 4°C. The cells then were resuspended in sterile water to an OD600 of 1.0. Serial 10-fold dilutions were spotted on the YNB agar plates using a replica plater. The agar plates were incubated at 30°C for 3 days and photographed. Representative images are shown. (TIF) [file pone.0093322.s004.tif]

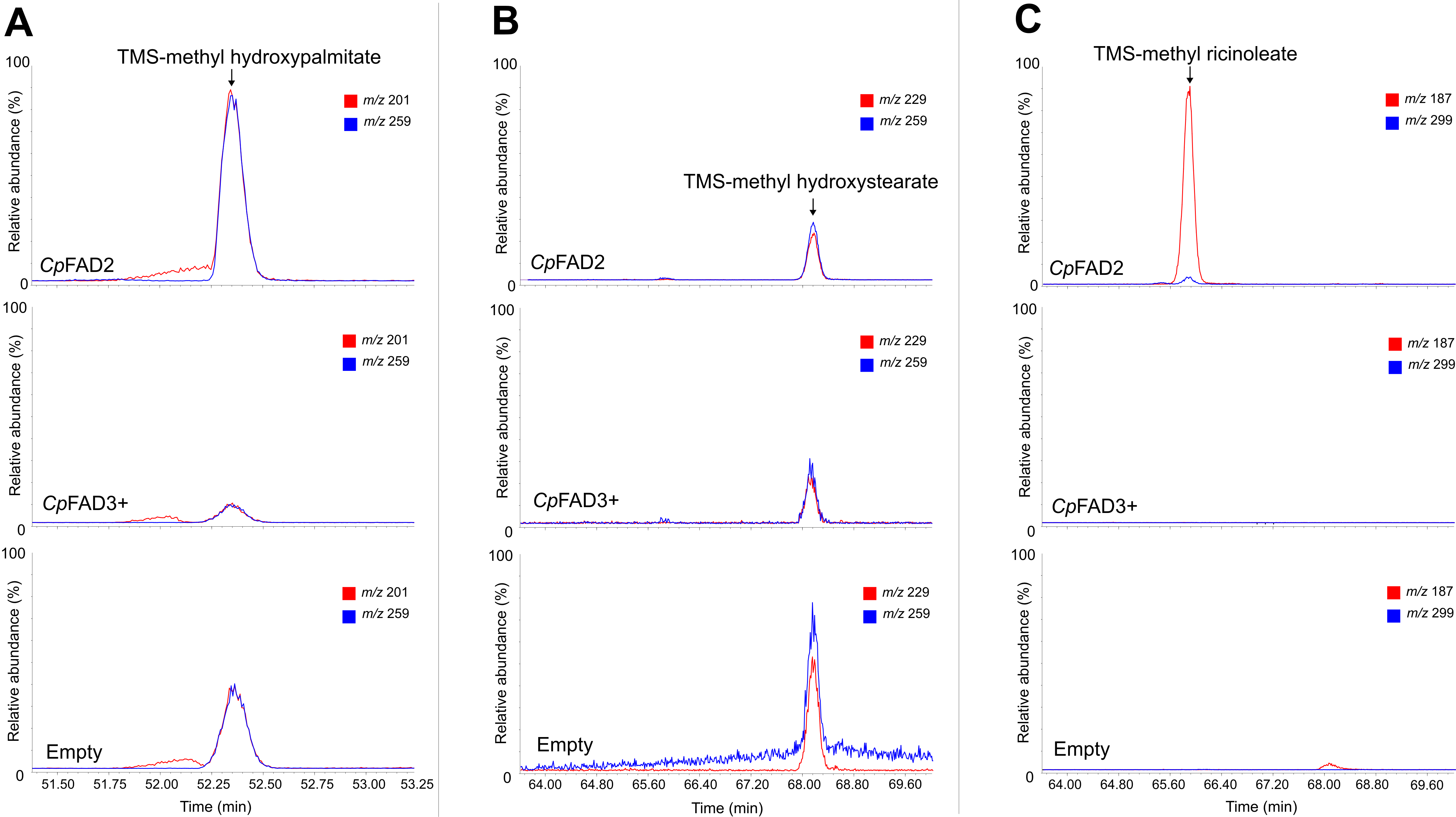

Supplement: Figure S5 — Extracted ion chromatograms of TMS derivatives of hydroxy FAMEs. TMS derivatives of FAME extracts from the CpFAD2 strain, CpFAD3 strain supplemented with linoleic acid and Empty strain are displayed in ion chromatograms extracted at m/z values characteristic for individual TMS-hydroxy FAMEs. (A) Ion chromatograms extracted at m/z 201 and 259 characteristic for TMS-methyl hydroxypalmitate, (B) ion chromatograms extracted at m/z 229 and 259 characteristic for TMS-methyl hydroxystearate and (C) ion chromatograms extracted at m/z 187 and 299 characteristic for TMS-methyl ricinoleate. (TIF) [file pone.0093322.s005.tif]
